# Internal structure and classification of pelvic floor dysfunction distress by PFDI-20 total score

**DOI:** 10.1186/s41687-022-00459-6

**Published:** 2022-05-16

**Authors:** Guilherme Tavares de Arruda, Dalton Francisco de Andrade, Janeisa Franck Virtuoso

**Affiliations:** 1 Departament of Physical Therapy, Universidade Federal de São Carlos, Rod. Washington Luiz, s/n, São Carlos, SP CEP: 13565-905 Brazil; 2 Departament of Technology Sciences, Universidade Federal de Santa Catarina, Florianópolis, Brazil; 3 Department of Health Sciences and Technology, Universidade Federal de Santa Catarina, Araranguá, Brazil

**Keywords:** Validation study, Patient-reported outcome measures, Pelvic floor disorders, Women

## Abstract

**Purpose:**

To evaluate the internal structure (structural validity and internal consistency) and propose a classification for the distress caused by the presence of pelvic floor dysfunction (PFD) symptoms based on the total score of the Pelvic Floor Distress Inventory (PFDI-20).

**Methods:**

Cross-sectional study conducted with Brazilian women over 18 years of age. Exploratory and confirmatory factor analysis were performed with Parallel Analysis and to test three models to compare them with the Root Mean Square Error of Approximation (RMSEA) and Comparative Fit Index (CFI). Internal consistency was calculated using Cronbach's alpha. Partial credit model (PCM) was performed to classify the total score of the PFDI-20.

**Results:**

Data from 237 women (49.62 ± 16.95 years) were analyzed. The one-dimensional structure had 43.74% of the explained variance with *α* = 0.929. The one-dimensional model was the most appropriate (CFI = 0.987 and RMSEA = 0.022). The total PFDI-20 score was classified as the absence of symptoms (score zero), symptoms with mild distress (1 to 15 points), symptoms with moderate distress (16 to 34 points), and symptoms with severe distress (35 to 40 points).

**Conclusion:**

The PFDI-20 has an one-dimensional structure and the distress caused by the presence of PFD symptoms can be classified as mild, moderate and severe. Health professionals and future studies can use our classification to facilitate the understanding of the patient's health status and to obtain other analyses on the severity of the distress of the symptoms of PFD.

## Plain English summary

There are limitations regarding the meaning of the total score of the Pelvic Floor Distress Inventory (PFDI-20) in clinical practice and scientific research. Thus, the aims of this study were to evaluate the internal structure (structural validity and internal consistency) and propose a classification for the distress caused by the presence of pelvic floor dysfunction (PFD) symptoms from the total score of PFDI-20. Cross-sectional study conducted with Brazilian women over 18 years of age. Data from 237 women (49.62 ± 16.95 years) were analyzed. The PFDI-20 has one-dimensional structure and the distress caused by the presence of PFD symptoms can be classified as mild, moderate and severe.

## Introduction

Patient-reported outcome measures (PROMs) are often assessed using questionnaires. PROMs are common in scientific research and clinical practice, since they are non-invasive, easy to apply, and low-cost methods [1]. However, to assess the measurement properties of a PROM it is necessary to verify if it is suitable for the construct and population that it intends to measure [2]. For this assessment, the COnsensus-based Standards for the selection of health Measurement INstruments (COSMIN) produces updated guidelines of wide use for the evaluation of PROMs from different areas of knowledge [2].

In women's health, the Pelvic Floor Distress Inventory (PFDI-20) [3] is a PROM that is often used in clinical practice and clinical trials to assess the distress caused by the presence of pelvic floor dysfunction (PFD) [4–6]. This PROM is advised as a grade A recommendation by the International Consultation on Incontinence (ICI) for clinical practice [7] and was translated and validated into several languages [8], including Brazilian Portuguese, with adequate values for test–retest reliability (intraclass correlation coefficient—ICC ≥ 0.70) and internal consistency (*α* ≥ 0.70) [6]. However, the structural factor of PFDI-20 has not been assessed in the Brazilian population, [8] and only one study showed results of its structural validity in Chinese women [9]. In this sense, structural validity is the degree to which the scores of a PROM are an adequate reflection of the dimensionality of the construct to be measured [2].

PFDI-20 assesses the distress of pelvic organ prolapse (POP), anorectal and urinary symptoms in three subscales, respectively, Pelvic Organ Prolapse Distress Inventory (POPDI-6), Colorectal-Anal Distress Inventory (CRADI-8) and Urinary Distress Inventory (UDI-6) [3]. According to the PFDI-20 development study, the calculation for assessing the distress of PFD symptoms was based on the Classic Test Theory (CTT), which takes into account the instrument as a whole and does not assess items or symptoms separately [3]. In addition, the PFDI-20 total score and each subscale is interpreted as the higher the score, the worse the distress [3]. This can affect the patient's comprehension of the meaning of the score for their health status and also limit statistical analysis in scientific research to only continuous data.

According to COSMIN, when an instrument does not have its consolidated factorial structure in which few studies have found different numbers of factors, or the method used for structural analysis is not clear, it becomes necessary to explore factorability by exploratory factor analysis (EFA) and then use confirmatory factor analysis (CFA) to confirm the adequacy of the results to the factors [2]. Due to this, it is necessary to assess the measurement properties of PFDI-20, especially the structural validity, in order to test its dimensionality and internal consistency, and also if this PROM is suitable for use in clinical practice in gynecology and scientific research. In addition, one of its strengths is the comprehensive assessment of the distress caused by the presence of PFD symptoms of three groups of PFDs in women. However, there are no established values for the classification of the distress intensity [3].

Obtaining values for the classification of the PFDI-20 total score will make it possible to classify the severity of the distress as mild, moderate and severe. For clinical practice, this may contribute to the assessment and reassessment of the patient after clinical or physical therapy treatment. For scientific research, it will be possible to classify the distress to allow a more comprehensive analysis of this problem and its impact on other variables. It is important for health professionals to identify the patients' health status more clearly and specifically. Thus, the aims of this study were to assess the internal structure (structural validity and internal consistency) of PFDI-20 in Brazilian women, according to the COSMIN guideline [2], and propose a classification for the distress caused by the presence of PFD symptoms from the PFDI-20 total score in mild, moderate and severe.

## Methods

Cross-sectional study approved by the Institutional Ethics Committee under No. 3,437,754. Permission was requested from the developer of the instrument, M.D. Matthew D. Barber, to use the PFDI-20. The data in this study come from a larger cross-sectional study “OMITTED FOR BLINDED PURPOSES”, which aims to assess the factors associated with PFD in women living in the south of Brazil.

Data collection was carried out between November 2019 and March 2020, and was interrupted due to the Coronavirus (COVID-19) pandemic. Brazilian women over 18 years old who went to the health units during the data collection period were included. Women who reported symptoms of urinary tract infection in the last week (pain and burning sensation when urinating), pregnant women, bedridden and those with low observable or self-reported cognitive ability to respond to the survey instruments were excluded. All women were invited to participate in the study, regardless of having diagnosis of PFD or not.

Data collection instruments consisted of the PFDI-20 and a characterization sheet, containing sociodemographic information, as well as gynecological and obstetric issues. PFDI-20 was translated and validated into Brazilian Portuguese by Arouca et al. [6] and assesses the distress of PFD symptoms in women. This instrument consists of three subscales, POPDI-6, CRADI-8 and UDI-6, with their own scores that assess the distress caused by the presence of POP, anorectal and urinary symptoms, respectively. The score for each subscale is calculated by averaging the sum of the items and the number of items in the subscale, and multiplying the result by 25. The PFDI-20 total score is represented by the sum of the scores of the three subscales, in which higher values indicate greater distress caused by the presence of PFD symptoms [3].

The data collection instruments were applied in the form of individual interviews by trained interviewers in a private place where the participants were approached. We chose to apply the instruments during an interview to avoid missing data. Sample size calculation followed the COSMIN guideline for studies on structural validity [2]. For factor analysis, the minimum sample size of 7 times the number of items in the instrument to be validated and greater than 100 individuals is advisable [2]. Therefore, for this study, a minimum sample of 140 women was appropriate, since the PFDI-20 has 20 items.

In the data analysis, a low frequency of “it bothers quite a bit” responses in the PFDI-20 items was observed through descriptive analysis. Because of this, the response categories “it doesn't bother at all” and “it bothers somewhat” were grouped in “with symptoms that don’t bother at all or bother somewhat”, and the answers “it bothers moderately” and “it bothers quite a bit” were grouped in “with symptoms that don’t bother moderately or bother quite a bit”.

Structural validity was assessed by EFA and CFA. For EFA, the Kaiser Meyer-Olkin index (KMO) and the Bartlett Sphericity Test were used to assess the factorability of the data. KMO values > 0.9 are considered excellent [10] and *p-*value < 0.05 in Bartlett's Sphericity Test indicates the factorability of the data [11]. The Diagonally Weighted Least Squares (DWLS) extraction method and a polychoric matrix were implemented in the analysis of the EFA. Parallel Analysis with random permutation of the observed data was used to decide the number of factors to be retained [12] and Robust Oblimin was used as a rotation method [13]. To check the adequacy of the model, the unidimensionality indicators Unidimensional Congruence (UniCo) and Mean of Item Residual Absolute Loadings (MIREAL) were used [14]. Values of UniCo > 0.95 and MIREAL < 0.30 indicate that the observed model can be treated as one-dimensional. The stability of factors was analyzed using the *H* index, which assesses whether a set of items represents an ideal common factor. *H* values range from 0 to 1, in which *H* ≥ 0.70 considers the factor as well defined [15]. These analyses were performed using FACTOR 10.10.02.

For CFA, three models were compared: one-dimensional, three-dimensional and five-dimensional. These models were based on the results of EFA and compared with results on the internal structure of PFDI-20 in the literature, assuming that the three subscales of PFDI-20 form 3 dimensions (POP, anorectal and urinary symptoms) and the five factors found by Ma et al. [9]. The models were compared using the Root Mean Square Error of Approximation (RMSEA), the Comparative Fit Index (CFI) and the Tucker-Lewis Index (TLI). RMSEA values less than 0.08 and CFI and TLI values above 0.90, or preferably, 0.95 are considered an adequate model fit [16]. For the selection of the most appropriate model, the Bayesian Information Criterion (BIC) factor retention method and Sample-Size Adjusted Bayesian (SABIC) were used. The model with the lowest BIC [17] and SABIC values was considered as having the most adequate factor structure [18]. Internal consistency, the degree of interrelation between the items [2], was calculated using the Cronbach's alpha for the model identified in the PFDI-20 EFA. Cronbach's alpha values ≥ 0.70 are considered acceptable [19]. These analyses were performed using JASP 0.14.1.

After the CFA, we were able to select the appropriate item response theory (IRT) model for the data, which was the partial credit model (PCM), introduced by Masters [20], considering the unidimensionality of the scale. Furthermore, the PFDI-20 items are ordinal polytomous and there is one-to-one relationship between the scores generated by classical test theory (CTT) and PCM [20]. This property allows the use of the CTT score to determine the respondent's latent trait (*ϴ*) level [21].

PCM is represented by the expression: $P_{i,k} \left( {\theta_{j} } \right) = \frac{{{\text{exp}}[\mathop \sum \nolimits_{u = 0}^{k} \left( {\theta j - bi,u} \right)]}}{{\mathop \sum \nolimits_{u = 0}^{{m_{i} }} {\text{exp}}[\mathop \sum \nolimits_{v = 0}^{u} \left( {\theta j - bi,v} \right)]}}$, in which $P_{i,k} \left( {\theta_{j} } \right)$ is the probability of a respondent choosing the category “*k*” (*k* = 0, 1, 2, …, *m*_*i*_), among the item categories$(m_{i} + 1)$; $b_{i,k}$ is the item parameter associated with the probability of the respondent choosing category “*k*” in item “*i*”. Further details on the PCM and methods for estimating item parameters and *ϴ* measurement can be obtained in the study by Andrade, Tavares and Cunha [21].

From the responses to the items obtained through the application of PFDI-20, the parameters of the items were estimated on a measurement scale (0–1), in which 0 is the mean of the *ϴ* of the participants and 1 is the standard deviation. With these values, the measure of the *ϴ* of each participant was estimated on the same scale. This allows the items to be positioned on the scale in order to enable their interpretation in the context of the *ϴ* measured. Then, each item/category was positioned at the point on the scale at which the likelihood that a participant, with a *ϴ* measurement equal to the point on the scale considered, would respond to a particular category of the item was ≥ 0.60 [21]. PCM analyses were performed using the *Rstudio* software 2021.09.1 and package *mirt*.

## Results

Two hundred and eighty women were selected to participate in the study, 43 (15.36%) of whom were excluded according to the eligibility criteria. At the end, the data of 237 (84.64%) women were analyzed. Table 1 shows the characteristics of the participants included in the study. Most of the participants (49.62 ± 16.95 years old) were married (n = 99; 41.77%), white (n = 167; 70.46%), had had only vaginal delivery (n = 84; 35.44%) and had not undergone a gynecological surgical procedure (n = 158; 66.67%). Most women did not report symptoms of stress urinary incontinence (SUI) (n = 185; 78.06%), urge urinary incontinence (UUI) (n = 158; 66.67%) and mixed urinary incontinence (MUI) (n = 204; 86.07%).

**Table 1:** Characterization of study participants (n = 237)

Characteristics	Mean ± SD or n (%)
Age (years)	49.62 ± 16.95
Years of study	11.10 ± 5.80
BMI (kg/m^2^)	27.77 ± 6.01
*Marital status*	
Married	99 (41.77)
Single	66 (27.85)
Divorced	24 (10.13)
Widow	30 (12.66)
Single, but lives with a partner	18 (7.59)
*Mode of delivery*	
Vaginal delivery only	84 (35.44)
Only cesarean delivery	64 (27.00)
Both mode of delivery	34 (14.35)
Nulliparous	55 (23.21)
*Skin color*	
White	167 (70.46)
Brown	46 (19.40)
Black	17 (7.17)
Indigenous	5 (2.11)
Asian	2 (0.84)
*SUI symptoms*	
No	185 (78.06)
Yes	52 (21.94)
*UUI symptoms*	
No	158 (66.67)
Yes	79 (33.33)
*MUI symptoms*	
No	204 (86.07)
Yes	33 (13.93)
*Gynecological surgical procedure*	
No	158 (66.67)
Yes	79 (33.33)

SD: Standard deviation. BMI: Body mass index. SUI: Stress urinary incontinence. UUI: Urge Urinary Incontinence. MUI: Mixed urinary incontinence

**Fig. 1:**
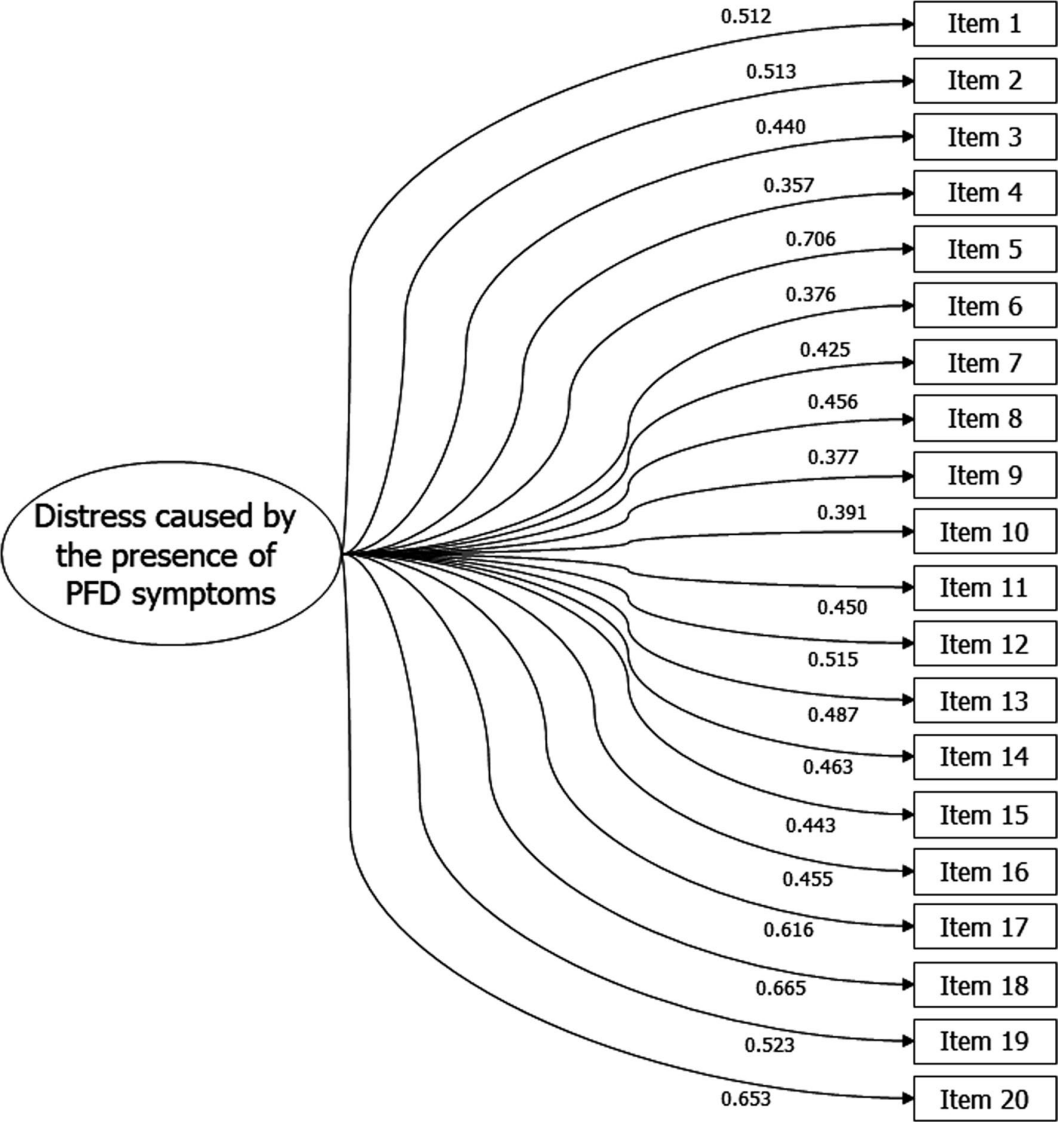
Path diagram of the one-dimensional model for PFDI-20. Pelvic Floor Dysfunction, PFD

Bartlett's sphericity tests (2630.7, *gl* = 190, *p* < 0.001) and KMO (0.912) suggested factorability of the items. Through the EFA, the parallel analysis suggested the one-dimensionality of the PFDI-20 as the most representative model for the data with 43.74% of the explained variance. The values of UniCo (0.966; 95%CI 0.944—1,000) and MIREAL (0.226; 95%CI 0.189—0.290) supported the hypothesis that the factorial structure of PFDI-20 can be considered one-dimensional. Table 2 and Fig. 1 show the high factor loadings of the one-dimensional model items (> 0.30). The Cronbach's alpha value of the one-dimensional model was considered adequate (*α* = 0.929).

**Table 2:** Factor loads of the one-dimensional structure of the PFDI-20

Items	Factorial load
1. Pressure in the lower abdomen	0.512
2. Heaviness or dullness in the pelvic area	0.513
3. Feeling of something coming out in the vaginal area	0.440
4. Push something in the vagina or to complete the evacuation	0.357
5. Incomplete emptying of the bladder	0.706
6. Push something around the vagina to complete the urination	0.376
7. Force or effort to evacuate	0.425
8. Incomplete emptying of the intestine	0.456
9. Involuntary loss of solid stools	0.377
10. Involuntary loss of liquid stools	0.391
11. Involuntary loss of gases	0.450
12. Pain during bowel movement	0.515
13. Urgency to evacuate	0.487
14. Sensation of rectal prolapse	0.463
15. Increased urinary frequency	0.443
16. Loss of urine during urgency	0.455
17. Loss of urine during laughing, coughing or sneezing	0.616
18. Loss of small amount of urine (drops)	0.665
19. Difficulty emptying the bladder	0.523
20. Pain in the lower abdomen or genital region	0.653
Variance explained	43.74%
Cronbach's alpha	0.929

Table 3 shows the values of the replicability measures of the factorial structure of the models. In all models, except for the fourth factor of the five-dimensional model, the *H*-latent values (*H* ≥ 0.70) suggest that the factors can be well identified by the response variables. The H-observed values of the one-dimensional model and the first factor of the five-dimensional model are those with ideal values (*H* ≥ 0.70). However, the other models suggested that the factors may not be replicable in other studies (*H* < 0.70).

**Table 3:** Factor replication measures of the PFDI-20 models

	One-dimensional model	Three-dimensional model			Five-dimensional model				
Factors		F1	F2	F3	F1	F2	F3	F4	F5
H-latent	0.936	0.821	0.927	0.886	1.077	0.892	0.822	0.787	0.917
H-observed	0.702	0.557	0.624	0.637	0.747	0.638	0.513	0.445	0.577

The comparison between the models by the CFA is shown in Table 4. The adjustment indices showed adequate values for the three models tested (CFI > 0.95, TLI > 0.95 and RMSEA < 0.08). After adjusting for the residual covariance of items 4, 7, 8, 9, 17 and 18 of the one-dimensional model, this model had lower BIC (7596.874) and SABIC (7460.579). However, CFI (0.752), TLI (0.718) and RMSEA (0.101) had low values.

**Table 4:** Factor adjustment and retention indexes for PFDI-20 models

Models	χ^2^ (df)	CFI	TLI	RMSEA (CI95%)	BIC	SABIC
One-dimensional	190.034 (170)	0.987	0.986	0.022 (0.000–0.038)	7781.409	7654.623
Three dimensions	144.695 (167)	0.997	0.996	< 0.001 (0.000–0.014)	7691.633	7555.338
Five dimensions	59.000 (100)	0.999	1.000	< 0.001 (0.000–0.204)	7631.770	7485.966
One-dimensional ajusted	570.830 (167)	0.752	0.718	0.101 (0.092–0.110)	7596.874	7460.579

*χ*^*2*^: chi-square. df: degrees of freedom. CFI: Comparative Fit Index. TLI: Tucker-Lewis Index. RMSEA: Root Mean Square Error of Approximation. BIC: Bayesian Information Criterion. SABIC: Sample-size Adjusted Bayesian

When comparing CTT with PCM, the items 3 (sensation of something coming out of the vaginal area), 4 (need to push something in the vagina or around the anus for complete evacuation), 6 (need to push something up in the vaginal area to start or complete the urination), 9 (anal incontinence—AI, solid stool), 10 (AI, liquid stool) and 14 (rectal prolapse symptom) presented only parameter *b1* (item difficulty) because their categories were grouped into two categories. It is possible to observe that there is a high probability that the participants with *θ* ≤  − 1.25 do not have symptoms of PFD (Appendix 1). Furthermore, the values calculated by the sum of the scores in all items ranged from 0 to 40 points. In this comparison, score 0 is equivalent to the absence of symptoms, scores from 1 to 15 points are equivalent to having most symptoms with mild distress, scores from 16 to 34 points are equivalent to having most symptoms to moderate distress, and scores to 35 to 40 points is equivalent to having symptoms with severe distress. Appendix 2 shows the new score for each item of PFDI-20.

According to our classification of the PFDI-20 score for the sample of this study, 182 (76.79%) women were classified as having symptoms and mild distress; 27 (11.39%) women had symptoms and moderate distress; 24 (10.13%) women had no symptoms; and 4 (1.69%) women were classified as having symptoms and severe distress.

## Discussion

In this study, we evaluated the internal structure of PFDI-20 in Brazilian women according to the COSMIN, and propose a classification for the distress caused by the presence of PFD symptoms assessed by the PFDI-20 total score. According to EFA, the PFDI-20 had one-dimensional structure, also presenting better indexes and an acceptable Cronbach's alpha. However, the model one-dimensions adjusted had better BIC and SABIC. In one-dimensional structure, all items of the instrument evaluate a single construct, the distress caused by the presence of PFD symptoms in women, differently from the factor structure found in a previous study [9]. Through the PCM, the proposed new PFDI-20 score classified the intensity of distress caused by the presence of PFD symptoms as mild (1—15 points), moderate (16—34 points) and severe (35—40 points). In our clinical experience, we believe that patients can better understand the severity of their own health problem through this new classification. Furthermore, it is possible that the perception and management of the distress caused by the presence of PFD symptoms are guided in different ways by clinicians for each severity.

In the literature, the factor structure of PFDI-20 was evaluated only by Ma et al. [9] in 126 Chinese women. In the same study [9], 5 factors were found that explained 69.55% of the variance: anal and colorectal distress (factor 1); direct POP feelings and symptoms of irritation or obstruction of the lower urinary tract (factor 2); various types of urinary incontinence (UI) (factor 3); external force to defecate (factor 4); and symptoms of rectocele (factor 5). In addition, the items of each subscale of PFDI-20 were combined in different factors and no EFA was performed. Considering the study by Ma et al., [9] the first to assess structural validity, it would be important to start the analysis of the factor structure by the EFA to explore the behavior of the items in the internal structure [22]. In contrast to the study by Ma et al. [9], in the present study we performed the EFA to identify the factorial structure of the data and the CFA to confirm its values according to the tested models, which is considered more appropriate when evaluating the factorial structure of a PROM [22].

All PFDI-20 items had acceptable factor loads (> 0.30), with high values (> 0.60) for items 5, 17, 18 and 20, which demonstrates that these items can distinguish more individuals with greater distress caused by the presence of PFD symptoms than items with lower factor loads. Compared to the study by Ma et al. [9], another important aspect of the PFDI-20 factorial structure found in this study was the application of the instrument in different cultures, which can be seen in the replicability values. In the present study, the replicability analysis showed that the one-dimensional model can be replicable in other populations. On the other hand, the models of three and five factors suggested that the factors may not be replicable in other populations. This means that future studies may find similar one-factor structures for PFDI-20, indicating a stability of the instrument in other populations.

Comparing the measure of the *ϴ* generated by the IRT with the score generated by the CTT, it was possible to classify the distress caused by the presence of PFD symptoms assessed by the PFDI-20 total score. Unlike CTT, IRT determines the probability of a participant selecting a response on the scale corresponding to their symptoms at a certain level of severity, i.e., in this study, "with mild symptoms and distress". In addition, the PCM assumes that respondents with more severe distress symptoms may be more likely to report symptoms that reflect worse distress severity [23]. Thus, items 3, 4, 6, 9, 10 and 14 of the PFDI-20 had a lower frequency of “bother moderately” and “bother quite a bit” responses, identifying participants with a high probability of presenting PFD symptoms that don’t bother at all or bother somewhat at *θ* ≥ 3.25. The low frequency of responses in these items may be due to the symptoms being considered more severe, because the sensation of something coming out of the vaginal area (item 3), the need to push something in the vagina or around the anus to complete the evacuation (item 4), need to push something around the vagina to complete the urination (item 6), AI (items 9 e 10) and rectal prolapse (item 14) are considered characteristic symptoms of greater severity among POP and anorectal symptoms [7, 24].

Through the observation of individuals and items on the same scale, it was possible to examine the relationship between an individual's response to each item and the response levels in the general *ϴ* that the item is intended to measure. In this case, the PCM provides information to discriminate individuals with different levels of *ϴ* and the category limit for each item [23]. Thus, the items or symptoms of PFDI-20 can be assessed according to their severity, and the classification of distress as mild, moderate and severe is feasible and easy to understand for both patients and clinicians.

PFDI-20 is often used to assess the distress caused by PFD and its symptoms in different populations [4, 9]. In this study, we included women with and without PFD, unlike other validation studies of PFDI-20, in which only women with PFD participated [3, 6, 9]. Thus, the greater variability of characteristics in this sample is understandable, which is evident in countries with diverse ethnicities and cultures such as Brazil. Furthermore, although the factorial structure of PFDI-20 is considered one-dimensional, according to a study not yet published in this sample, the items can still assess different symptoms of PFD. Thereby, we emphasize that the use of this PROM in scientific research or clinical practice must consider the symptoms assessed in each item for each PFD, and also thoroughly evaluate the distress caused by the presence of PFD symptoms in women.

In this study, we employed adequate methods for obtaining the factorial structure of a set of items from PFDI-20 and followed the COSMIN [2] for the best quality of evidence in validation studies. We also used appropriate methods for obtaining the total score of a set of items from PFDI-20 [20, 21]. In addition, our proposal for classifying the distress caused by the presence of PFD symptoms by PFDI-20 is simple, easily applicable and understandable, which may contribute to its widespread use in clinical practice and scientific research. However, the selection of a convenience sample and the lack of an evaluation of medical diagnoses for PFD can be considered limitations of this study.

## Conclusion

PFDI-20 can be organized in a one-dimensional structure for assessing the distress caused by the presence of PFD symptoms and has acceptable internal consistency. Furthermore, the distress caused by the presence of PFD symptoms can be classified as mild, moderate and severe, according to PFDI-20 total score

## Data Availability

Data are available from the main author.

## Appendix 1

See Table

**Table 5:** Scale and positioning of the PFDI-20 items according to the PCM and CTT

		Scale													
PCM		− 1.5	− 1	− 0.5	0	0.5	1	1.5	2	2.5	3	3.5	4	4.5	
CTT		0	1 to 15						16 to 34		35 to 40				
Item	Parameters														
1	b1	1.79							0.63	0.80	0.90	0.96	0.98	0.99	1.00
	b2	1.24								0.54	0.70	0.82	0.89	0.93	0.96
2	b1	2.26							0.58	0.76	0.89	0.95	0.98	0.99	1.00
	b2	0.83								0.58	0.75	0.86	0.92	0.95	0.97
3	b1	2.74										0.57	0.68	0.78	0.85
4	b1	3.22											0.57	0.69	0.78
5	b1	1.37						0.55	0.73	0.86	0.93	0.97	0.99	1.00	1.00
	b2	1.22								0.59	0.73	0.83	0.90	0.94	0.96
6	b1	4.17													0.58
7	b1	1.35						0.64	0.80	0.91	0.96	0.98	0.99	1.00	1.00
	b2	0.56							0.58	0.73	0.84	0.91	0.94	0.97	0.98
8	b1	0.89						0.66	0.81	0.90	0.96	0.98	0.99	1.00	1.00
	b2	1.25								0.61	0.74	0.84	0.90	0.94	0.96
9	b1	3.50												0.62	0.73
10	b1	2.99											0.62	0.73	0.82
11	b1	1.90							0.65	0.81	0.91	0.96	0.99	0.99	1.00
	b2	0.93								0.61	0.76	0.86	0.92	0.95	0.97
12	b1	2.09							0.65	0.82	0.92	0.97	0.99	1.00	1.00
	b2	0.63								0.65	0.80	0.88	0.93	0.96	0.98
13	b1	0.72					0.57	0.74	0.86	0.94	0.97	0.99	1.00	1.00	1.00
	b2	0.89							0.56	0.70	0.81	0.88	0.93	0.96	0.97
14	b1	2.93											0.64	0.75	0.83
15	b1	0.88						0.66	0.80	0.90	0.96	0.98	0.99	1.00	1.00
	b2	1.30								0.60	0.73	0.83	0.89	0.93	0.96
16	b1	2.44							0.65	0.82	0.92	0.97	0.99	1.00	1.00
	b2	0.20								0.70	0.84	0.91	0.95	0.97	0.98
17	b1	1.74						0.64	0.81	0.92	0.97	0.99	0.99	1.00	1.00
	b2	0.00							0.66	0.81	0.89	0.94	0.97	0.98	0.99
18	b1	1.37						0.63	0.80	0.91	0.96	0.98	0.99	1.00	1.00
	b2	0.59							0.57	0.73	0.84	0.90	0.94	0.97	0.98
19	b1	2.92								0.65	0.82	0.92	0.97	0.99	1.00
	b2	0.72									0.70	0.84	0.91	0.95	0.97
20	b1	2.84							0.52	0.73	0.87	0.95	0.98	0.99	1.00
	b2	0.37								0.61	0.78	0.88	0.94	0.97	0.98

PFDI-20: Pelvic floor distress inventory. PCM: Partial credit model. CTT: Classical test theory. b1 and b2: difficulty parameters of the items. Values of parameters b1 and b2 < 0.5 were removed from the table for better visualization

5.

## Appendix 2

See Table.

**Table 6:** Distribution of the scores for the PFDI-20 response categories

		Responses	
Items	No	Yes, it doesn't bother at all or somewhat	Yes, it bothers moderately or quite a bit
1	0	1	2
2	0	1	2
3	0	2	2
4	0	2	2
5	0	1	2
6	0	2	2
7	0	1	2
8	0	1	2
9	0	2	2
10	0	2	2
11	0	1	2
12	0	1	2
13	0	1	2
14	0	2	2
15	0	1	2
16	0	1	2
17	0	1	2
18	0	1	2
19	0	1	2
20	0	1	2
Variation of the total PFDI-20 score: 0—40			
Classification: 0 (no symptoms and no distress), 1—15 points (with symptoms and mild distress), 16—34 (with symptoms and moderate distress), 35—40 (with symptoms and severe distress)			

PFDI-20: Pelvic floor distress inventory

6
